# Online tracking of the contents of conscious perception using real-time fMRI

**DOI:** 10.3389/fnins.2014.00116

**Published:** 2014-05-23

**Authors:** Christoph Reichert, Robert Fendrich, Johannes Bernarding, Claus Tempelmann, Hermann Hinrichs, Jochem W. Rieger

**Affiliations:** ^1^Department of Neurology, University Medical Center A.ö.R.Magdeburg, Germany; ^2^Department of Knowledge and Language Processing, Otto-von-Guericke UniversityMagdeburg, Germany; ^3^Forschungscampus STIMULATEMagdeburg, Germany; ^4^Department of Psychological and Brain Sciences, Dartmouth CollegeHanover, NH, USA; ^5^Institute for Biometry and Medical Informatics, Medical Faculty, Otto-von-Guericke UniversityMagdeburg, Germany; ^6^Department of Behavioral Neurology, Leibniz Institute for NeurobiologyMagdeburg, Germany; ^7^German Center for Neurodegenerative Diseases (DZNE)Magdeburg, Germany; ^8^Center for Behavioral Brain SciencesMagdeburg, Germany; ^9^Department of Applied Neurocognitive Psychology, Carl-von-Ossietzky UniversityOldenburg, Germany; ^10^Research Center for Neurosensory Sciences, Carl-von-Ossietzky UniversityOldenburg, Germany

**Keywords:** anorthoscopic, bistable perception, ambiguous stimulus, real-time fMRI, object integration, slit viewing

## Abstract

Perception is an active process that interprets and structures the stimulus input based on assumptions about its possible causes. We use real-time functional magnetic resonance imaging (rtfMRI) to investigate a particularly powerful demonstration of dynamic object integration in which the same physical stimulus intermittently elicits categorically different conscious object percepts. In this study, we simulated an outline object that is moving behind a narrow slit. With such displays, the physically identical stimulus can elicit categorically different percepts that either correspond closely to the physical stimulus (vertically moving line segments) or represent a hypothesis about the underlying cause of the physical stimulus (a horizontally moving object that is partly occluded). In the latter case, the brain must construct an object from the input sequence. Combining rtfMRI with machine learning techniques we show that it is possible to determine online the momentary state of a subject's conscious percept from time resolved BOLD-activity. In addition, we found that feedback about the currently decoded percept increased the decoding rates compared to prior fMRI recordings of the same stimulus without feedback presentation. The analysis of the trained classifier revealed a brain network that discriminates contents of conscious perception with antagonistic interactions between early sensory areas that represent physical stimulus properties and higher-tier brain areas. During integrated object percepts, brain activity decreases in early sensory areas and increases in higher-tier areas. We conclude that it is possible to use BOLD responses to reliably track the contents of conscious visual perception with a relatively high temporal resolution. We suggest that our approach can also be used to investigate the neural basis of auditory object formation and discuss the results in the context of predictive coding theory.

## Introduction

Human cognitive neuroscience makes the strong assumption that all subjective human experience is tightly linked to spatiotemporal patterns of a neuronal activation. For sensory perception this assumption predicts that patterns of brain activity should not only reflect the physical stimulus but also the content of perceptual awareness derived from the physical input (von Helmholtz, 1867). Perceptually ambiguous stimuli, in which the same physical stimulus elicits categorically different and mutually exclusive percepts, provide an excellent opportunity to investigate constructive brain processes underlying the formation and the maintenance of subjective perceptual experiences. The neural correlates of different ambiguous visual stimuli have been studied using fMRI, including binocular rivalry (Tong et al., 1998; Haynes and Rees, 2005), ambiguous static figures (Kleinschmidt et al., 1998), structure-from-motion (Brouwer and van Ee, 2007; Freeman et al., 2012), occluded moving drawings (Murray et al., 2002; Fang et al., 2008), and moving plaids (Castelo-Branco et al., 2002). It has been suggested that a strong link between dynamically changing brain activation patterns and subjective perceptual states can be established by predicting different states of perception from brain activation (Cox and Savoy, 2003; Rieger et al., 2008; Chang et al., 2010). However, none of the previous fMRI-studies on perceptual organization of ambiguous stimuli made a serious attempt to quantify the relevance of the observed BOLD-modulations for the tracking of spontaneously changing states of awareness.

**Figure 1:**
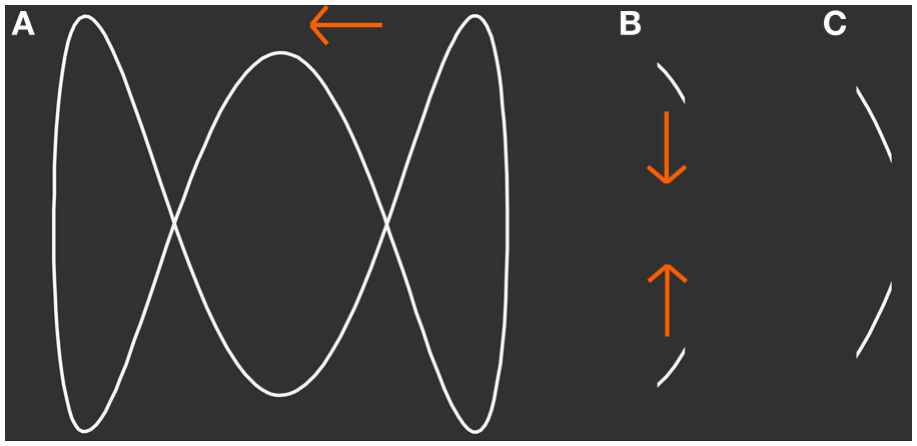
**Presented stimulus. (A)** The shape of a 3-loop-figure that was simulated to move horizontally back and forth behind a narrow aperture. **(B,C)** Show snapshots of the figure that can be seen through the simulated aperture at two different points in time. Although only line segments are visible at each point in time the subject is able to perceive the figure as a whole. At a specific width of the aperture the participant's conscious perception switches spontaneously between vertically jumping line segments and the horizontally moving figure. From **(B)** to **(C)** the figure moves to the left.

In vision, occlusion of a moving object, a situation common in everyday vision, requires integration of sequentially visible object fragments into the percept of a coherent object. The stimulus we employ in this study, mimics this occlusion problem (Figure 1). It simulates an outline figure moving horizontally back and forth behind an occluder with a narrow vertical aperture, a situation similar to looking through a door that is only open a slit. Importantly, with our stimulus an observer's conscious percept intermittently switches between the actually presented sequence of object parts, short line segments moving vertically, and a horizontally moving integrated object that is located behind an occluder. In previous studies (Fendrich et al., 2005; Rieger et al., 2007) we have shown that under free viewing conditions the integrated object percept is constructed post-retinally in the brain and that eye movements do not contribute to the construction of the integrated object. Murray et al. (2002) investigated the effects of dynamic bistable figure integration on the BOLD-activation level in human V1. The subjects observed a diamond shaped outline figure moving behind apertures. The authors reported a decrease of BOLD-activation in V1 while subjects perceived the integrated figure and conclude that this reduction is in concordance with predictive coding theory. Predictive coding theory states that representation of simple stimulus features in early sensory cortices is explained away by feedback from higher areas involved in the creation of derived percepts, such as occluded objects. Fang et al. (2008) and de-Wit et al. (2012) reported activation changes in the lateral occipital complex (LOC) with opposite sign than those in V1. Unfortunately, all these authors reported only BOLD-modulations in a small pre-selected set of visual brain areas, and therefore do not address the extent and functional characteristics of the brain networks that carry information about the content of visual perception and are thus likely to be involved in establishing the current percept of the ambiguous stimulus.

In the auditory domain, object formation from sequentially presented tones requires temporal integration, similar to the occluded-object stimulus we are using in the current study. Whole head fMRI suggests roles for intraparietal sulcus in modality independent structuring of sensory input Cusack (2005) and feedforward-feedback interactions between cortical and thalamic regions during percept switching (Kondo and Kashino, 2009). In addition, neuromagnetic measurements indicate that non-primary auditory areas maintain the representation of auditory streams (Gutschalk et al., 2005). Furthermore, left auditory cortex was found to play a dominant role in active auditory stream segregation (Deike et al., 2010). Interestingly, Pressnitzer and Hupé (2006) suggest that although in vision and audition object formation and perceptual bistablity may have different underling neuronal correlates, both modalities may share similar functional mechanisms with similar dynamics. Thus, a method suitable to establish a tight link between BOLD-activity and the state of awareness in bistable visual object formation will likely be useful to investigate bistable auditory object formation.

Our first goal in this study is to determine if the brain activation changes linked to the two conscious percepts of our ambiguous display are robust enough to allow a reliable determination of the current content of conscious visual perception. It should be noted that activation changes that occur exclusively in the visual system will not necessarily be the most informative indicators of the actual conscious visual percept. Therefore, we use whole-brain fMRI scans and multivariate machine learning techniques to determine brain activation patterns that discriminate between subjective perceptual states (Norman et al., 2006). As an ultimate proof of concept our main goal was to perform the discrimination online. In our asynchronous online prediction scheme no prior knowledge about the time of percept switches is available which makes tracking of percepts more challenging than prediction of synchronous perceptual events (e.g., Hollmann et al., 2011). To date, real-time fMRI has been employed in several tasks to predict brain states (LaConte et al., 2007; Hollmann et al., 2011) and to provide online feedback (Weiskopf et al., 2007; Weiskopf, 2012). However, we are unaware of a study that shows the feasibility of rtfMRI discrimination of the contents of visual perception with ambiguous stimuli. Furthermore, we propose a method to test the generalizability of the multivariately assessed brain patterns over subjects. Our third goal was to characterize the brain networks that allow for discrimination between the perception of integrated objects vs. object parts and to establish whether the relative activation levels in this network are concordant with predictive coding theory. The methods we apply may also be applicable for discriminating bistable auditory percepts, provided that effect sizes and temporal dynamics are similar.

## Materials and methods

### Stimuli and stimulus presentation

The stimulus was the simulation of an outline loop-figure moving horizontally behind a narrow vertical aperture with invisible borders (Figure 1). It was back projected onto a translucent screen with a JVC DLA-G150CL projector and viewed by the subject via a mirror. The eye-screen distance was 59 cm.

The width of the aperture was individually adjusted such that observers had intermittent percepts that spontaneously switched between the physical stimulus, short line segments with changing orientation moving vertically up and down, or an integrated but occluded object moving horizontally back and forth behind an aperture (Fendrich et al., 2005). The height and width of the outline figure was 6.8 and 8.7° visual angle, respectively. The individual aperture widths ranged between 3.7 and 7.0% of the outline figure width. We will denote the percept of the physical stimulus as the lines percept and the percept of the integrated figure as the object percept.

Subjects freely viewed the display and reported their percept by holding one of two buttons pressed throughout the whole interval the percept lasted. We found in earlier investigations that under free viewing conditions eye movements tracking the horizontal object movements were negligibly small and did not influence the quantity or quality of object percepts (Fendrich et al., 2005; Rieger et al., 2007). The assignment of the button presses to the line and object percepts was switched after each run to avoid a correlation of any purely motor BOLD-activations produced by the button presses with the activations produced by changes in the conscious percepts. In real-time mode, we presented visual feedback by switching line color slightly and auditory feedback by presenting the decoded percept as spoken word from an audio file in the moment of switch detection, respectively.

### Subjects and experiments

Data were acquired in two experiments which we will refer to as offline experiment and online experiment. In the offline experiment, we used conventional fMRI to record the BOLD-activations while subjects viewed the stimulus and reported their subjective percepts via button presses. Fifteen subjects participated in this experiment (8 female, 7 male, mean age = 26.3 years). The Data of this experiment we analyzed only in an offline mode as it is conventionally performed in fMRI. In order to prove the reliability of our approach, we conducted a second experiment. The online experiment was designed to track the current content of conscious perception using real-time fMRI BOLD-measurements, and we performed both an online and offline data analysis. Ten subjects participated in this experiment (6 female, 4 male, mean age = 25.7 years).

The experiments were approved by the ethics committee of the Medical Faculty of the Otto-von-Guericke University. All subjects gave their informed consent prior to the start of the experiment. They had normal or corrected to normal vision, and were paid for participation.

### MR-scanning

A Siemens-Trio scanner equipped with an 8-channel phased array head-coil was used for anatomical and functional MRI. Full head T1-weighted anatomical images were obtained with an MPRAGE sequence (80 axial slices, slice thickness = 2 mm, field of view = 256 by 192 mm; inplane matrix = 256 by 192). Functional images were obtained from the whole head with a gradient recalled echoplanar imaging (EPI) sequence [32 axial slices, slice thickness = 4 mm, field of view = 200 mm; inplane matrix = 64 by 64; *TR* = 2.5 s (offline experiment) and 2.0 s (online experiment), echo time = 30 ms (offline experiment) and 27 ms (online experiment)]. Each run lasted 420 s, and each subject completed between 7 and 8 runs in the offline experiment and 8 runs in the online experiment.

### Support vector machine learning

Support vector machines (SVMs) represent a class of machine learning algorithms characterized by good generalization performance even in high dimensional feature spaces (Vapnik, 1998). This is achieved because the internal regularization avoids overfitting so that the complexity of the classifier does not depend on the complexity of the feature space (Cherkassky and Mulier, 1998). Therefore, SVMs are a suitable approach for analyzing fMRI signals, particularly when a whole head analysis is preferred. The central idea of the algorithm is to maximize the distances from the training data to the separating hyperplane which is characterized by its normal vector $\vec{w}$, also referred to as the weight vector, and a bias parameter *b*. With these parameters estimated from the training data, labels *y*_*i*_ of new data $\vec{x}$_*i*_ can be predicted by a simple inner product with the function:

(1) $$y_{i}=sign\left(\vec{w}\cdot {\vec{x}}_{i}+b\right)$$

Note that the inner product between the classifier weights and the measured data can be interpreted as a weighted “voting” of each voxel for one or the other class. Due to the properties of the inner product, votes for positive and negative classes can be generated by multiple combinations of positive or negative weights with positive or negative voxel values. It is thus difficult to interpret the sign of the weight. A comprehensive introduction to support vector machine learning is provided in Burges (1998). In our study we pre-selected a relatively high number of voxels and assigned the signal change of each voxel as input data for a linear SVM. Labels for both training and testing of the SVMs were derived from the button presses shifted 5 s forward in time to account for the delay of the hemodynamic response function (HRF).

### Data processing

#### Preprocessing

In the offline discrimination of line vs. object percepts we employed several preprocessing steps using the SPM5 package (Wellcome Department of Cognitive Neurology, University College London, UK). We corrected slice acquisition time and head movements, and we spatially normalized the individual brains to the MNI standard brain. Finally, we spatially smoothed the functional data with an 8 mm FWHM Gaussian kernel. In the temporal domain we band-pass filtered the voxel time series. We set the high-pass cut-off frequency to 1/128 Hz and determined the low-pass cut-off frequency between 1/27 and 1/8 Hz individually as described below. We discarded voxels exceeding 10% signal change from the analysis because at 3T they are likely to stem from large vessel contributions.

#### Offline-classification

In the offline analysis which was applied to both experiments, we performed a leave-one-run-out cross-validation rather than using a leave-one-volume-out or random-selection of volumes approach to test for generalizability. This avoids information transfer between training and test set due to temporal correlations in the slowly varying BOLD response. In this procedure, all runs but one are used for feature selection, classifier training, and filter optimization. The decoding accuracy of the trained classifier is then tested on the reserved test set.

We used a univariate statistical approach for feature selection to reduce computational costs. Therefore, we calculated model BOLD-responses from the reported object and line percepts, regressed them on to the voxel BOLD-time courses and calculated univariate *F*-tests to assess statistical difference between the BOLD-amplitudes estimated for the two percepts. Finally, the 5000 voxels with the lowest *p*-value were selected for the classification step.

In addition to feature selection, the cutoff frequency of the low-pass filter was optimized in the cross-validation loop to minimize high frequency noise and to account for individual variations in the duration of the percepts. We tested the classification performance on the training set with four low-pass cutoff frequencies 1/27, 1/18, 1/12 and 1/8 Hz and selected the cut-off with the smallest classification error in cross validation performed on the training set. Although minimal loss on the training set does not necessarily imply optimal performance on the test data, we found this approach appropriate.

The trained classifier was then applied to each EPI-volume in the left out test run to determine the subject's current conscious percept from the BOLD-activation patterns. For evaluation of the classifier's discrimination performance we compared the time course of the subject's button presses shifted by 5 s to take into account the HRF delay. For classification and cross-validation we combined the Princeton MVPA toolbox (http://code.google.com/p/princeton-mvpa-toolbox/) with the Spider machine learning toolbox (http://www.kyb.tuebingen.mpg.de/bs/people/spider).

#### Online-classification

Online analysis is the ultimate test of the ability to track the phenomenal content of ambiguous perception time resolved from BOLD-activations. We implemented a real-time fMRI-classification experiment based on the experimental description language EDL (Hollmann et al., 2008). Data acquisition was performed with the same parameters as in the offline experiment except for shorter TRs. The MR scanners' EPI scanning protocol was modified to immediately export the acquired and motion corrected volumes (Hollmann et al., 2008). On the classification host, transformation matrices for spatial normalization were calculated from the first acquired EPI-volume and applied to all subsequently acquired volumes. After normalization, the EPI-volumes were spatially smoothed (8 mm FWHM Gaussian kernel). Stimulus presentation and classification were performed on two separate computers that communicated via RS-232 connection.

The first two runs of each scanning session were used for feature selection and for training of the initial classifier. First, the BOLD time series were zero centered and the baseline values were kept for subsequent subtraction to approximate a zero centered signal online. Then the time series were temporally filtered applying a digital fourth order butterworth band-pass filter with cutoff frequencies 1/128 and 1/16 Hz. Initial univariate feature selection was performed similar to the procedure described in the offline experiment except that we applied an ANOVA as statistical test and retained the voxels with the smallest 10,000 *p*-values for a first pass of classifier training. We used the initial classifier for a second, multivariate feature selection in which we retained only voxels with feature weights exceeding a threshold *w*_*th*_ which was defined at *w*_*th*_ = 10^−1^*max*|*w*_*k*_|, *k* = 1 … 10,000. The resulting feature space was used for further analysis including retraining on the current data set. Online discrimination of the content of perception started with the third run. Auditory feedback was provided by playing the respective word (female voice speaking) when a switch in the state of the percept was detected. Furthermore, the according state was continuously indicated by slightly changing the color of line segments to greenish for decoded object percepts and to reddish for decoded line percepts. Analogous to the offline experiment, subjects reported their perceptual state by button presses. Moreover, we instructed the participants to mentally assess the correctness of the decoded percept, but to consider a delay of approximately 8 s, expectable due to the hemodynamic response delay and data processing time. Eight runs were acquired for each subject. The classifier was updated after every run starting from the third.

#### Cross-subject generalization

In analogy to the classical statistical approach, we aimed to use multivariate classification to derive predictive patterns of BOLD-activation that would generalize across individual subjects. Multivariate pattern analysis often focuses on individual discriminative patterns within a small circumscribed brain area (e.g., Kay et al., 2008). Therefore, this approach is often not commensurate to the attempt in classical statistical analysis to generalize experimental results to a population. Our approach aims to derive patterns of BOLD-activation from a group of subjects that will generalize to new subjects. The generalization is tested by showing that the patterns found can discriminate conscious perceptual contents with better than chance performance in data from new subjects. This last test of generalization performance is typically omitted in classical statistics but critical for the evaluation of the relevance of brain activation patterns at the population level, as it is performed with standard parametric univariate testing.

In our approach, we used a leave-one-subject-out cross-validation. Because the total number of EPI-volumes (19,188 in the offline experiment and 16,480 in the online experiment, respectively) is prohibitively large for our whole head analysis approach and considering that classification appears to be more reliable distant from perceptual switches, we reduced the amount of training data by averaging over three EPI-volumes around the center of each interval of a sustained percept. This reduced the data set to 2526 EPI-volumes in the offline experiment and 1412 EPI-volumes in the online experiment, respectively.

For feature selection we trained an SVM on all available volumes of a single subject involving 30,000 voxels with lowest *p*-values derived from an *F*-statistic. Then we calculated a weighted combination of each training subject's weight vector

(2) $${\bar{w}}^{\;k}=\frac{1}{n}\sum_{i\;=\;1}^{n}2\left({\hat{p}}_{i}-\frac{1}{2}\right)w_{i}^{k}$$

In this equation, *w*^*k*^_*i*_ is the individual weighting of the *k*^th^ feature in the normal vector $\vec{w}$_*i*_ and $\hat{p}$_*i*_ is the rate of correct classifications achieved with the *i*^*th*^ of *n* subjects. The result is a map of voxels weighted by both the importance of the voxel for the individual subjects and the relevance of the whole individual voxel map for the discrimination of the perceptual content. To perform the group analysis, we generated these maps in cross-validations and selected the 30,000 highest weighted voxels from the combined map.

#### Permutation testing

We evaluated the reliability of the classifier's discrimination rate with respect to the empirical guessing level and its 95% confidence obtained with a permutation test. Only discrimination rates exceeding the 95% confidence interval for guessing were considered reliable (Good, 2005). Note that the mean empirical guessing levels can substantially deviate from the expected level (50% in the two class case), and may have wide confidence intervals (see e.g., Rieger et al., 2008). For permutation testing, the available labels, line, or object percept, were randomly reassigned among the measured EPI-volumes. Ideally, this procedure should prevent the classifier from learning information useful for separating the classes. However, even though labels are randomly assigned to the EPI-volumes in most cases the classifier will discriminate line and object percepts with a rate different from 50% correct. The critical question is, whether the discrimination rate obtained with the actually measured data set is within the confidence interval for discrimination rates that can be obtained with randomly assigned labels. However, since we discriminate events in a continuous time series the permutation scheme should consider temporal correlations due to the distribution of durations of the line and object percepts. We retained these durations in the permutation samples by permuting full blocks of consecutively equal sample labels. The empirical guessing levels were estimated within the above described cross-validation framework in a total of 500 random permutations.

A second application of permutation testing is to generate a distribution of classifier weights that can be obtained by separating the data without knowledge of actual class labels. We used this method to determine the significance that a feature weight deviates from a randomly obtained weight. According to a Mourão-Miranda et al. (2005) we calculated the *p*-value, denoted *p*_*w*_, as the ratio of number of features exceeding the weight of the classifier in the distribution of randomly obtained weights for this feature to the number of permutations. A low value for *p*_*w*_ indicates that the voxel contributes discriminative information to the classifier.

## Results

### Behavioral results

**Figure 2:**
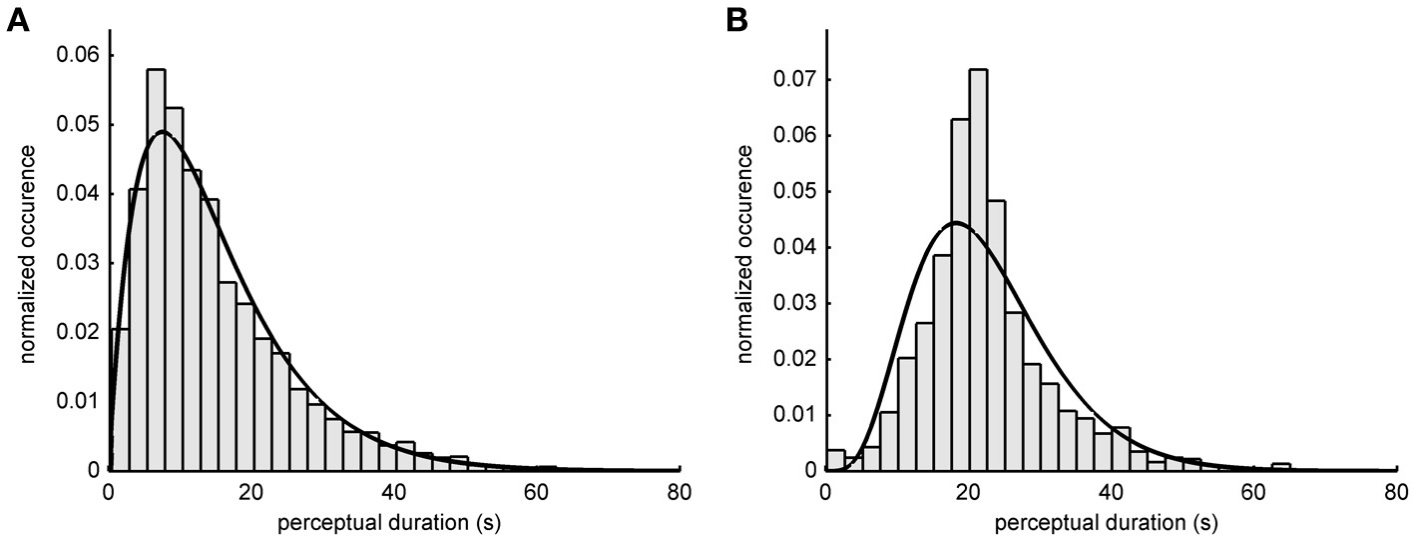
**Perceptual switch intervals**. Histograms show the relative occurrence how long a perceptual state lasted for **(A)** 15 participants of the offline experiment and **(B)** 10 participants of the online experiment. A gamma probability density function (pdf) was fitted to the distributions. The median duration of one percept in the offline experiment was 12.1 s (pdf shape: 2.00; scale: 7.51). In the online experiment the median duration of the percept was 21.4 s (pdf shape: 5.29; scale: 4.26).

The distribution of the duration of the perceptual interval lengths in bistable percepts can be approximated by a gamma probability density function (pdf) (Kleinschmidt et al., 1998). Figure 2 shows the histograms of the interval durations in the two experiments and the fitted gamma pdf. The median duration in the online experiment was 21.4 s (Figure 2B). The maximum of the fitted gamma pdf is at 18.3 s (goodness of fit: *R*^2^ = 0.8416). In the offline experiment the median interval duration was 12.1 s (Figure 2A) and the maximum of the gamma pdf is at 6.9 s (goodness of fit: *R*^2^ = 0.9851). The longer perceptual intervals in the online experiment were presumably due to familiarizing the subjects with the stimulus before the beginning of the experiment. The average proportion of object percepts does not differ between the online and the offline experiment [offline: 53.9%, online: 51.7%, two-sample *t*-test: *t*_(23)_ = 0.9, *p* = 0.37].

### Accuracy of the decoded percepts

**Figure 3:**
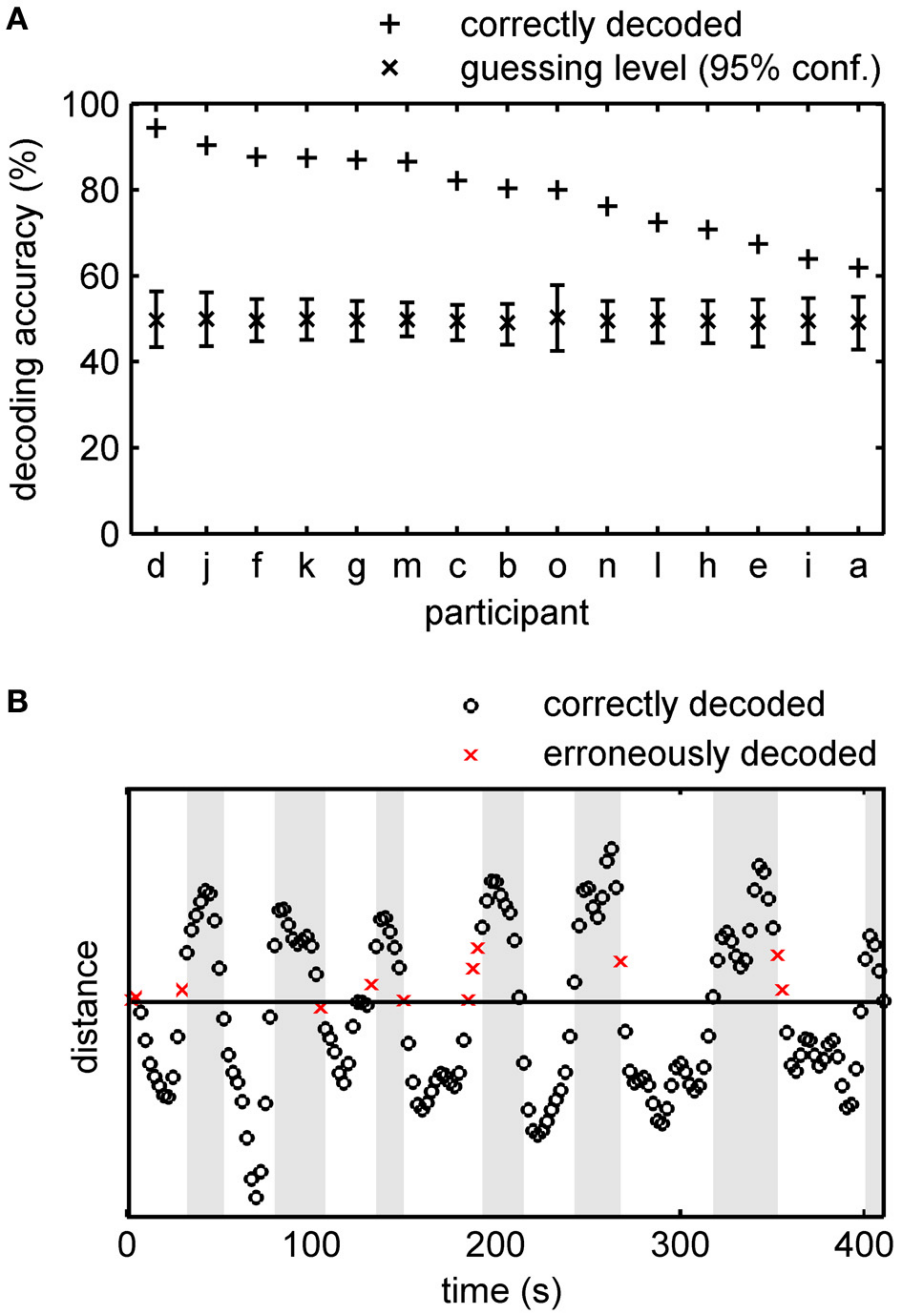
**Accuracy of offline tracking. (A)** Single subject decoding accuracy obtained by a leave-one-run-out cross-validation. On average 79.2% (SE: 2.6%) of the acquisition time the decoded percept was correct. For each participant the mean guessing level as determined by permutation testing is approximately 50% and the fraction of correctly decoded perceptual states exceeds the 95% confidence interval of the guessing level. Participants are sorted by performance. **(B)** Time course of the distance (arbitrary unit) of the data points in classification space to the separating hyperplane constructed by a SVM for participant *d*, Run 5 (92.7% accuracy). White background signifies intervals in which the participant indicated perceiving the integrated object and gray background signifies intervals with line percepts. EPI-volumes for which the conscious percept was correctly identified are shown as circles, incorrectly identified percepts as crosses.

Averaged over all subjects, the phenomenal content of perception (lines or object) was correctly determined 79.2% of the time (EPI-volumes) in the offline experiment, with a standard error of the mean (SE) of 2.6%. Figure 3A shows the individual results for all 15 subjects including the empirical 95% confidence intervals for guessing. In the best subject, the classifier tracked the phenomenal content nearly perfectly, at 94.4%. Most errors in this subject occurred in EPI-volumes around the time of a perceptual switch (Figure 3B). This observation was also found in other subjects and is supported by the observation that without the three volumes around a perceptual switch the decoding accuracy over all subjects increases to 84.8% and that the decoding accuracy dropped to 72.4% when we considered only the EPI-volumes around the switches for accuracy calculation. This suggests that the limited temporal sampling of fMRI (here 0.4 Hz sampling rate) may contribute to the decoding error. Importantly, in every subject the classification rate clearly exceeds the 95% confidence interval for guessing. The theoretical 50% guessing level is always included in the 95% confidence interval determined by the permutation test. This indicates that there is no bias for one class in the data sets (Rieger et al., 2008).

### Online tracking of conscious perception

Online, time resolved discrimination of the phenomenal content of the subjects' percepts was possible with great precision. Importantly, the online prediction started after only 14 min of training data acquisition (412 samples). On average 82.8% (SE: 2.4%) of the time the classifier correctly determined whether the subject currently perceived lines or an integrated object. Classification rates ranged from nearly perfect (94.2%) for the best subject to 65.9% for the worst. Although the classifier was retrained after each run, decoding accuracies did not significantly change over runs (average linear regression slope −0.24%, *p* > 0.05). In concordance with the offline experiment, most discrimination errors appeared around the perceptual switch (average decoding accuracy increases to 87.0% correct when ignoring three volumes around switch and decreases to 70.4% correct when taking only switch volumes into account), indicating that a considerable proportion of the errors might occur due to slow hemodynamic response and a relative low sampling rate of fMRI.

The average temporal delay from the beginning of the EPI-volume acquisition to feedback was 4.3 s (SE: 0.05 s). About half of this delay was due to the EPI-acquisition (*TR* = 2 s). Most of the remaining delay was required for data transfer and signal processing. Assuming a hemodynamic delay of 5 s, feedback was delayed by 9.3 s. The median duration of a phenomenal percept was 21.4 s and only 4.5% of the percept durations were shorter than this delay. Subjects reported that they could readily relate the feedback to their phenomenal percepts despite the delay. The feature selection scheme described in section Online-classification revealed an average feature set size of 3997 voxels (SE: 215 voxels).

**Figure 4:**
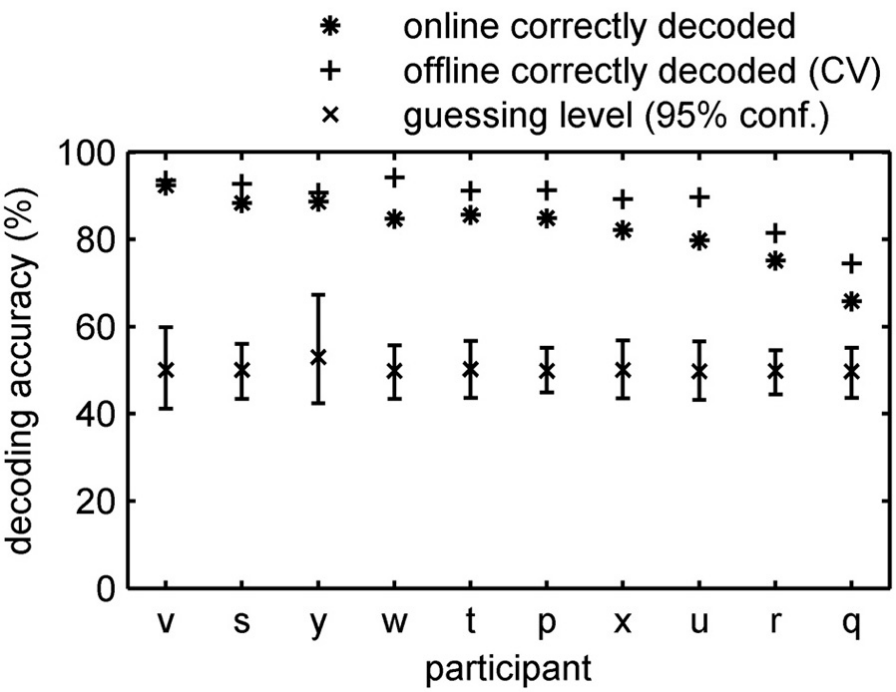
**Accuracy of online tracking**. Single subject decoding accuracy obtained during the online decoding experiment with feedback. On average 82.8% (SE: 2.4%) of the time the content of the conscious percept was correctly decoded. An additional offline cross-validation procedure applied to the same data revealed 88.8% (SE: 1.9%) accuracy on average. Participants are sorted by performance.

The classification rates obtained in an online experiment are most likely sub-optimal. One reason is that we stopped feature selection after the second run. To test if even more reliable information about the momentary conscious percept can be revealed by optimization of the signal processing chain we performed an additional offline leave-one-run-out cross-validation analysis in which we optimized preprocessing parameters as described in section Offline-classification. On average the decoding accuracy increased by 6% to an accuracy of 88.8% (SE: 1.9%; 74.5–94.2%) (Figure 4). All classification rates clearly exceeded the individually determined 95% confidence intervals for guessing. Importantly, the decoding accuracy is higher when subjects received feedback compared to the experiment without feedback presentation. Our results clearly show that fMRI in combination with advanced machine learning approaches makes it possible to track the phenomenal content of a subject's percept online with substantial time resolved accuracy.

### Generalization over subjects

In a final analysis we investigated whether a classifier derived from a population of subjects, would be capable of predicting the phenomenal content of a new subject's percept. This is of particular interest for brain computer interface related research. We trained the classifier in a leave-one-subject-out cross-validation scheme as described in section Cross-subject generalization. When we excluded the EPI-volumes around perceptual switches for training and evaluation, using only the EPI-volumes with the most reliable labels, we obtained 78.1% correct classifications for the data from the offline experiment and 79.4% accuracy for the data from the online experiment. Including the EPI-volumes acquired around switches in the evaluation yielded 68% accuracy with the data from the offline and 73.11% with the data of the online experiment. In this analysis accuracy exceeded the guessing level for all but one of the subjects. The result suggests that a classifier derived from a population can indeed transfer to new subjects, although information loss occurred compared to the single subject analysis.

### Brain areas involved in percept discrimination

An essential question is what brain networks discriminate between the contents of conscious percepts in dynamic object integration. We analyzed the trained classifiers to address this question. In case the input features are scaled comparably, it is possible to interpret the feature weights learned with linear SVM as informative voxels in a brain network that discriminates the contents of the percepts.

**Figure 5:**
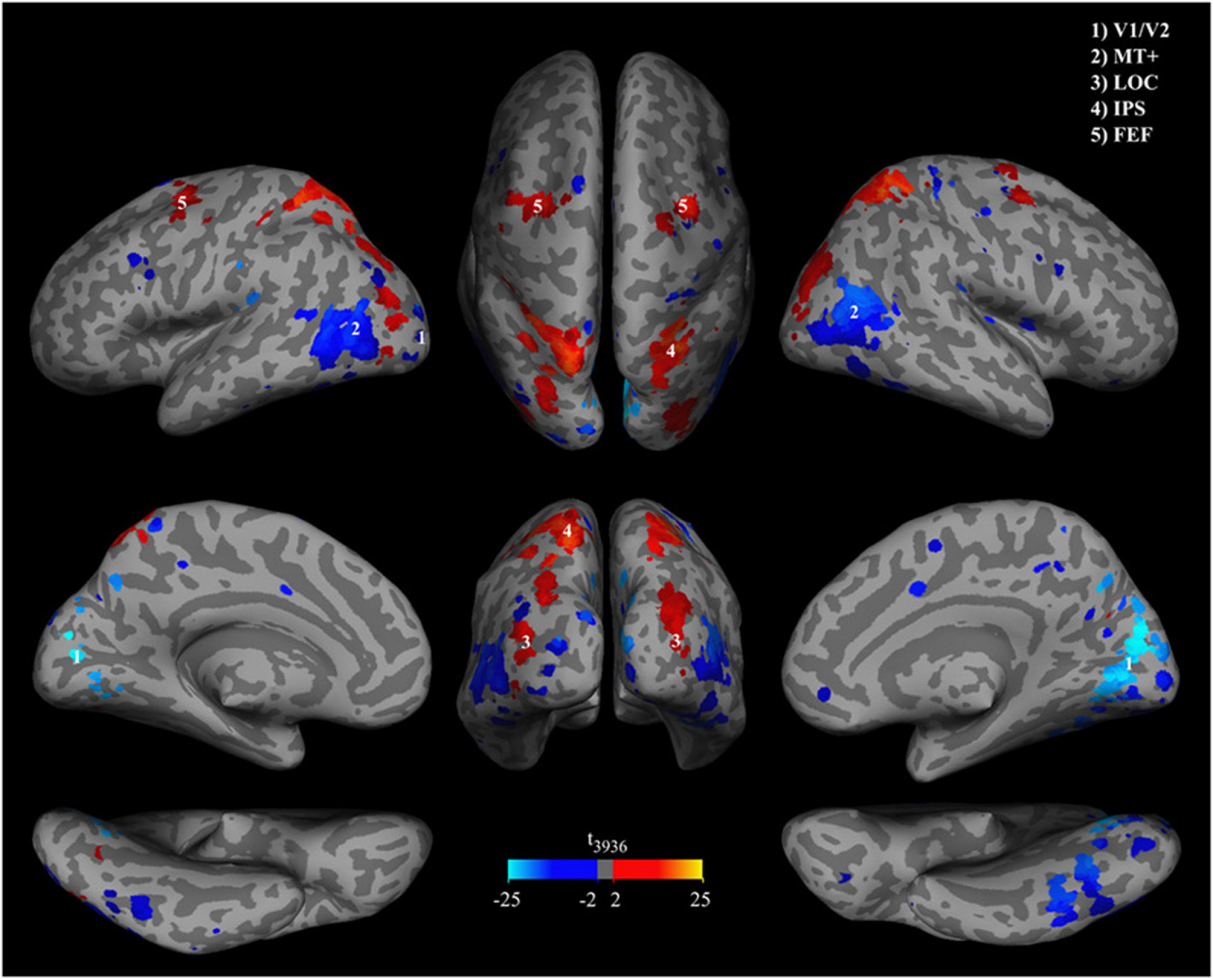
**Significance of classifier weights and activation differences**. Brain patterns were extracted from the group classifiers of both offline and online experiments and overlaid on an MNI template. Brain regions were masked by *p*-values of classifier weights, thresholded at **p_w_** < 0.1. *P*-values were obtained in a permutation test. Hot colors indicate higher BOLD signal during object percepts and cool colors indicate lower BOLD signals during object percepts.

To determine if a voxel contributed significantly to discrimination of percepts we used a combined multivariate and univariate approach. First we calculated a multivariate *p*-value *p*_*w*_ for each voxel by non-parametric permutation testing involving all subjects from both experiments using the group data sets described in Cross-subject generalization and labels “line” or “object” percept permuted among the BOLD-data. This procedure provides a null distribution of weights for each voxel. We trained 1000 classifiers for both group data sets. In the next step we used the weights from the classifiers trained with the actually measured labels and the permutation distributions of weights to calculate the probability *p*_*w*_ that the weights obtained with the “correct” labels were observed by chance. In Figure 5 we show a map of the voxels that reached a weight-threshold of *p*_*w*_ < 0.1 in the conjunction of both experiments. Second, in order to show BOLD change direction and univariate effect sizes we calculated *t*-values for each voxel. Red to yellow indicates that BOLD-activity in the voxel is higher during object percepts and blue to cyan indicates lower BOLD-activity during object percepts. The two sided *t*-value for a conventional univariate threshold of *p* < 0.001 would correspond to *t*_(3936)_< −3.2 or *t*_(3936)_> 3.2, which applies to 88.6% of the voxels shown in Figure 5 as well as to all of the clusters shown. Note that the maps are smooth and unvariate effects are uniform in each cluster. This smoothness indicates systematic effects over voxel in each cluster. Moreover, the combination of multivariate classifier training with univariate analysis suggests that the classifier does not only cancel out correlated noise in voxels with reliable weights.

Brain areas that contributed significantly to the classification include relatively early retinotopic visual cortex [MNI coordinates (5, −77, 8), *t*_(3936)_ = −23.0] and the middle temporal MT+ [MNI coordinates (47, −70, 13) and (−39, −65, 12), *t*_(3936)_ = −14.5 and *t*_(3936)_ = −13.7] where BOLD activity is lower during the object perception, and ventral visual stream areas such as putative lateral occipital complex [LOC, MNI coordinates (32, −88, 13) and (−34, −88, 8), *t*_(3936)_ = 4.6 and *t*_(3936)_ = 7.4] where BOLD activity is higher during the object percept. However, the whole head classification approach suggests that the discriminative network in most subjects includes brain areas that are not primarily visual such as the lateral intraparietal sulcus [IPS, MNI coordinates (27, −57, 63) and (−22, −58, 58), *t*_(3936)_ = 15.9 and *t*_(3936)_ = 14.3], the frontal eye fields [FEF, MNI coordinates (30, −9, 51) and (−28, −7, 53), *t*_(3936)_ = 7.5 and *t*_(3936)_ = 13.2], and sporadic small clusters of voxels in the frontal lobe.

## Discussion

In this work we show that it is possible to use rtfMRI to reliably track online the current content of conscious visual perception while observers view ambiguous stimuli. The reliability of the approach is indicated by permutation tests and the observation that errors occurred primarily close to the time of perceptual switches. Importantly, we found that with our approach the trained classifiers generalize over subjects. Our approach to investigating the neural networks that underlie perceptual representation is data driven as well as hypothesis generating. This distinguishes it from earlier approaches that have focused on hypothesis testing (Kleinschmidt et al., 1998; Murray et al., 2002; Yin et al., 2002; Haynes and Rees, 2005). Our approach revealed novel components of a network that is informative about the current content of visual perception and allowed the relevance of these components to be quantified in terms of their decoding accuracy.

### Linking subjective percepts with objective measurements of brain activation

There is a long standing debate as to whether subjectively perceived qualities can be related to objectively observed neural activations. Several decoding studies have successfully shown that certain stimulus features can be decoded from BOLD-activity (for reviews see Rees et al., 2002; Tong et al., 2006; Tong and Pratte, 2012). However, it is not always clear in these studies if informative changes in brain activation relate to encoding of visual features or subjective percepts. A particularly strong case is made for decoding of subjective awareness when changes in the subjective state are uncorrelated with the physical stimulus (e.g., Grill-Spector et al., 2000; Chang et al., 2010). Here we employed a visual stimulus that is either perceived as a pair of vertically moving lines or as an occluded object moving behind a narrow slit. Importantly, earlier studies (Fendrich et al., 2005; Rieger et al., 2007) demonstrated that eye movements did not contribute to the formation of the object percept. This implies that the changes in brain activation we tracked reflect brain processes related to changes in subjective experiences rather than changes in the physical stimulus. We show to our knowledge for the first time that spontaneous changes in subjective, stimulus independent experiences can be tracked online with high accuracy, good temporal resolution (0.5 Hz sampling rate), and over an extended time period (>1 h) using real time fMRI. The high decoding accuracies we obtained, with up to 94% correct predictions, accentuate the strength of the link between neural network states and states of subjective perception.

The informative patterns of brain activations we found with our approach generalized well over subjects. This is not generally the case and may depend on the spatial resolution of the attempted analysis (e.g., Haxby et al., 2001) or the measurement technique employed (Fazli et al., 2009). We found that functional MRI combined with normalization to a standard brain and slight smoothing is a successful strategy for revealing informative networks at the scale of the full brain that transfer well between subjects. Such networks likely reflect general mechanisms underlying perceptual representation. The relative importance of individual vs. general (between-subject) features of brain organization and brain processing strategies can be estimated by assuming that the accuracy obtained with an individual classifier reflects maximum obtainable performance, given a particular classification approach. The relative reduction in accuracy produced by the between-subject generalization then indicates the importance of individual features. In our investigation this reduction was statistically significant but almost all of the decoding accuracies achieved with left-out subjects were well above chance level. This is important because it indicates that patterns generalizing over subjects have a meaningful physiological interpretation. Together these results indicate that our data driven approach can be used in combination with ambiguous stimuli to reveal brain networks that are tightly linked to subjective percepts.

### Brain networks with decreased BOLD-activity during integrated object percepts

Analyzing the importance of individual brain voxels for the discrimination of the line vs. object percepts revealed an extended posterior brain network in which activation in lower tier sensory brain areas decreases during object percepts and activation in higher tier brain areas tends to increase. The results from our data driven approach confirm and considerably extend previous studies.

In concordance with earlier studies using similar ambiguous stimuli (Murray et al., 2002; Fang et al., 2008; de-Wit et al., 2012) we find a reduction of brain activity in early retinotopic visual areas, that represent the physical stimulus features such as line length and orientation. Concordant with de-Wit et al. (2012) we observe that these activation reductions extend around calcarine sulcus into the depth of the hemispheric cleft. In our study we also find reduced activation during object percepts in an area on the lateral surface, in the ascending limb of the inferior temporal sulcus, a reliable landmark for human motion sensitive area MT+ (Dumoulin et al., 2000) and in the vicinity of the fusiform gyrus, considered to be involved in higher level visual object representations (Ishai et al., 2000). Murray et al. (2002) argue that the reduction of activation in V1 during integrated object percepts is in accord with predictive coding theory (Mumford, 1992; Rao and Ballard, 1999). In this theory, higher tier areas convey a prediction of the features of the object represented to the lower tier areas which signal the difference between the prediction and the actual stimulus. This error signal is fed forward to the higher tier areas to adjust the object representation. The smaller the error between the prediction from higher tier areas and the stimulus representation in early sensory areas, the lower is their activation. Murray et al. (2002) suggested that during integrated object percepts higher tier brain areas predict the representation of the sensory stimulus in earlier areas and, as a consequence, the activation drops in lower tier areas. The reduced activation in hMT+ during object percepts fits with this interpretation. In earlier studies (Fendrich et al., 2005) we could show that during object percepts, the brain derives knowledge of horizontal object motion and could predict the movement of the integrated moving object from the movement of the segments. Similar to our results Castelo-Branco et al. (2002) found increased MT+ activity while the movement of two plaid stimuli was perceived as belonging to different objects and decreased activity when they were perceived as one integrated object. However, more detailed analysis reveals two effects that may require further elaboration of the predictive coding explanations of the neural deactivation patterns. First, we find reduction of brain activation during object percepts in ventral brain areas which are considered object selective and located relatively high in the processing hierarchy, according to standard theories of visual processing (Ungerleider and Mishkin, 1982). Conversely, within the predictive coding framework, the reduction of activation during object percepts suggests that these brain areas are located relatively low in the processing hierarchy. Second, as de-Wit et al., 2012 already pointed out, the reduction of activation in early visual cortex may extend beyond the retinotopic representation of the stimulus.

### Brain networks with increased BOLD-activity during integrated object percepts

In concordance with Fang et al. (2008) we find increased brain activity bilaterally on the lateral surface of the occipital lobe during object percepts. This brain area is localized in the anatomical region of the LOC, which has been linked to the construction of object shape (Kourtzi and Kanwisher, 2000). The other areas with increased information were located toward and in parietal cortex. The function of the parietal areas is less clear. Recent studies suggest a functional role of parietal areas in the grouping of sequentially presented elements into coherent object percepts. For example, Zaretskaya et al. (2013) reported increased activation in anterior intraparietal sulcus (aIPS) during grouping of local elements into objects, and Peltier et al. (2007) reported LOC and IPS activation during haptic shape perception in which the sequential haptic information about shape has to be integrated into a coherent object percept. These parietal activations are not unique to the visual modality. Cusack (2005), using an auditory streaming paradigm, reported increased activity in IPS during object integration and hypothesized that the intraparietal area is generally involved in structuring sensory information. Moreover, we consider it highly unlikely that the parietal activation patterns can be explained by eye movements alone. In two earlier studies (Fendrich et al., 2005; Rieger et al., 2008) we characterized eye movements during the line vs. object percepts using highly accurate eye tracking methods and found only a small difference in the eye movement patterns between lines and object percepts.

Together, the results from our data driven approach suggest a working hypothesis: the LOC together with parietal brain areas are involved in constructing the percept of the integrated object. The reduction of activation in early visual areas during object percepts might be an indication of an interaction between higher and lower tier brain areas in which the former predict the representations in the latter. However, the spatial extent of the activation reduction in early retinotopic cortex and the function of ventral object selective cortices in the visual hierarchy require further evaluation.

### Real time vs. offline fMRI-analysis

Real-time analysis allowed us to feed the decoded percepts back to the subject. For such biofeedback experiments it is necessary that the classification method provides high generalization performance and that it requires little training data. We found that the average accuracies in the online and offline experiments were comparable, with a slight tendency for higher accuracies in the online experiment. Theoretically, the online classification should perform worse than the cross-validation method because it uses a smaller training set. Subsequent cross-validation analysis of the online data confirmed that this offline approach can further increase the accuracy obtained with the online data set. We speculate that the reason of the accuracy advantage in the online experiment is due to the feedback which may have increased the subject's sense of agency. Receiving a feedback of the current conscious perceptual state could reduce mental fatigue and thereby reduce noise, which is essential for good classification performance. Another factor that may have contributed to the increased accuracy is the longer durations of the perceptual intervals which may be better reflected in the slow BOLD-response. The observation that most classification errors occurred around the time of perceptual switches supports this assumption. Studies indicate that subjects can volitionally control the switching of the percepts (van Ee et al., 2005; Kornmeier et al., 2009). Due to the delay of the BOLD-response it takes several seconds until the feedback signals a switch in perception. Although the subject's informal reports indicated that they could deal well with the delay, too short perceptual intervals could have led to more discordant feedback. This may have motivated our subjects to volitionally control and extend the duration of the percepts in order to increase the synchronization of the percept with the delayed feedback. Thus, the feedback may have increased perceptual inertia or cognitive bias leading to prolonged percepts in the online experiment. Further studies are necessary to characterize the neural basis of this biofeedback effect.

### Ambiguity and stream segregation

Ambiguous stimuli can induce bistable perceptual organization that switches between categorically different object percepts despite constant visual input. This property makes them ideal to investigate processes of perceptual organization independent of potential confounds by changing physical stimuli. Here we outlined a data driven classification approach to reveal brain networks underlying perceptual organization. The classification accuracy provides an intuitive measure of the relevance of the observed effects. We would like to point out, that the approach is not restricted to the visual domain. In the auditory domain, multistable stimuli, such as two tone sequences (Bregman, 1990; Gutschalk et al., 2005; Deike et al., 2010), play an important role in the characterization of processes underlying stream organization. We suggest that the approach presented here, can be used to investigate the neural basis of auditory object formation with ambiguous auditory stimuli. Support for the assumption that this is possible comes from studies showing that the dynamics of auditory and visual perceptual switches are similar. In both modalities percept durations are gamma distributed and have similar lengths (Pressnitzer and Hupé, 2006; Denham et al., 2012). Furthermore, the different modalities seem to share common brain regions for perceptual organization (Cusack, 2005).

### Conflict of interest statement

The authors declare that the research was conducted in the absence of any commercial or financial relationships that could be construed as a potential conflict of interest.

## References

[B1] Bregman A. S. (1990). Auditory Scene Analysis: The Perceptual Organization of Sound. Cambridge, MA: MIT Press

[B2] Brouwer G. J. van Ee R. (2007). Visual cortex allows prediction of perceptual states during ambiguous structure-from-motion. J. Neurosci. 27, 1015–1023 10.1523/JNEUROSCI.4593-06.2007 17267555 PMC6673188

[B3] Burges C. J. C. (1998). A tutorial on support vector machines for pattern recognition. Data Min. Knowl. Discov. 2, 121–167 10.1023/A:1009715923555

[B4] Castelo-Branco M. Formisano E. Backes W. Zanella F. Neuenschwander S. Singer W. (2002). Activity patterns in human motion-sensitive areas depend on the interpretation of global motion. Proc. Natl. Acad. Sci. U.S.A. 99, 13914–13919 10.1073/pnas.202049999 12368476 PMC129797

[B5] Chang E. F. Rieger J. W. Johnson K. Berger M. S. Barbaro N. M. Knight R. T. (2010). Categorical speech representation in human superior temporal gyrus. Nat. Neurosci. 13, 1428–1432 10.1038/nn.2641 20890293 PMC2967728

[B6] Cherkassky V. Mulier F. M. (1998). Learning from Data: Concepts, Theory, and Methods. New York, NY: John Wiley & Sons

[B7] Cox D. D. Savoy R. L. (2003). Functional magnetic resonance imaging (fMRI) brain reading: detecting and classifying distributed patterns of fMRI activity in human visual cortex. Neuroimage 19, 261–270 10.1016/S1053-8119(03)00049-1 12814577

[B8] Cusack R. (2005). The intraparietal sulcus and perceptual organization. J. Cogn. Neurosci. 17, 641–651 10.1162/0898929053467541 15829084

[B9] Deike S. Scheich H. Brechmann A. (2010). Active stream segregation specifically involves the left human auditory cortex. Hear. Res. 265, 30–37 10.1016/j.heares.2010.03.005 20233603

[B10] Denham S. Bendixen A. Mill R. Toth D. Wennekers T. Coath M. (2012). Characterising switching behaviour in perceptual multi-stability. J. Neurosci. Methods 210, 79–92 10.1016/j.jneumeth.2012.04.004 22525854

[B11] de-Wit L. H. Kubilius J. Wagemans J. Op de Beeck H. P. (2012). Bistable Gestalts reduce activity in the whole of V1, not just the retinotopically predicted parts. J. Vis. 12:12 10.1167/12.11.12 23090610

[B12] Dumoulin S. O. Bittar R. G. Kabani N. J. Baker C. L. Le Goualher G. Pike G. B. (2000). A new anatomical landmark for reliable identification of human area V5/MT: a quantitative analysis of sulcal patterning. Cereb. Cortex 10, 454–463 10.1093/cercor/10.5.454 10847595

[B13] Fang F. Kersten D. Murray S. O. (2008). Perceptual grouping and inverse fMRI activity patterns in human visual cortex. J. Vis. 8:2 10.1167/8.7.2 19146235

[B14] Fazli S. Popescu F. Danószy M. Blankertz B. Müller K. R. Grozea C. (2009). Subject-independent mental state classification in single trials. Neural Netw. 22, 1305–1312 10.1016/j.neunet.2009.06.003 19560898

[B15] Fendrich R. Rieger J. W. Heinze H. J. (2005). The effect of retinal stabilization on anorthoscopic percepts under free-viewing conditions. Vision Res. 45, 567–582 10.1016/j.visres.2004.09.025 15621175

[B16] Freeman E. D. Sterzer P. Driver J. (2012). fMRI correlates of subjective reversals in ambiguous structure-from-motion. J. Vis. 12:6 10.1167/12.6.35 22753440

[B17] Good P. I. (2005). Permutation, Parametric, and Bootstrap Tests of Hypotheses. New York, NY: Springer

[B18] Grill-Spector K. Kushnir T. Hendler T. Malach R. (2000). The dynamics of object-selective activation correlate with recognition performance in humans. Nat. Neurosci. 3, 837–843 10.1038/77754 10903579

[B19] Gutschalk A. Micheyl C. Melcher J. R. Rupp A. Scherg M. Oxenham A. J. (2005). Neuromagnetic correlates of streaming in human auditory cortex. J. Neurosci. 25, 5382–5388 10.1523/JNEUROSCI.0347-05.2005 15930387 PMC1237040

[B20] Haxby J. V. Gobbini M. I. Furey M. L. Ishai A. Schouten J. L. Pietrini P. (2001). Distributed and overlapping representations of faces and objects in ventral temporal cortex. Science 293, 2425–2430 10.1126/science.1063736 11577229

[B21] Haynes J. D. Rees G. (2005). Predicting the stream of consciousness from activity in human visual cortex. Curr. Biol. 15, 1301–1307 10.1016/j.cub.2005.06.026 16051174

[B22] Hollmann M. Mönch T. Mulla-Osman S. Tempelmann C. Stadler J. Bernarding J. (2008). A new concept of a unified parameter management, experiment control, and data analysis in fMRI: application to real-time fMRI at 3T and 7T. J. Neurosci. Methods 175, 154–162 10.1016/j.jneumeth.2008.08.013 18773922

[B23] Hollmann M. Rieger J. W. Baecke S. Lützkendorf R. Müller C. Adolf D. (2011). Predicting decisions in human social interactions using real-time fMRI and pattern classification. PLoS ONE 6:e25304 10.1371/journal.pone.0025304 22003388 PMC3189203

[B24] Ishai A. Ungerleider L. G. Martin A. Haxby J. V. (2000). The representation of objects in the human occipital and temporal cortex. J. Cogn. Neurosci. 12, 35–51 10.1162/089892900564055 11506646

[B25] Kay K. N. Naselaris T. Prenger R. J. Gallant J. L. (2008). Identifying natural images from human brain activity. Nature 452, 352–355 10.1038/nature06713 18322462 PMC3556484

[B26] Kleinschmidt A. Büchel C. Zeki S. Frackowiak R. S. J. (1998). Human brain activity during spontaneously reversing perception of ambiguous figures. Proc. Biol. Sci. 265, 2427–2433 10.1098/rspb.1998.0594 9921682 PMC1689542

[B27] Kondo H. M. Kashino M. (2009). Involvement of the thalamocortical loop in the spontaneous switching of percepts in auditory streaming. J. Neurosci. 29, 12695–12701 10.1523/JNEUROSCI.1549-09.2009 19812344 PMC6665088

[B28] Kornmeier J. Hein C. M. Bach M. (2009). Multistable perception: when bottom-up and top-down coincide. Brain Cogn. 69, 138–147 10.1016/j.bandc.2008.06.005 18682314

[B29] Kourtzi Z. Kanwisher N. (2000). Cortical regions involved in perceiving object shape. J. Neurosci. 20, 3310–3318 10777794 10.1523/JNEUROSCI.20-09-03310.2000 PMC6773111

[B30] LaConte S. M. Peltier S. J. Hu X. P. P. (2007). Real-time fMRI using brain-state classification. Hum. Brain Mapp. 28, 1033–1044 10.1002/hbm.20326 17133383 PMC6871430

[B31] Mourão-Miranda J. Bokde A. L. W. Born C. Hampel H. Stetter M. (2005). Classifying brain states and determining the discriminating activation patterns: support vector machine on functional MRI data. Neuroimage 28, 980–995 10.1016/j.neuroimage.2005.06.070 16275139

[B32] Mumford D. (1992). On the computational architecture of the neocortex. II. The role of cortico-cortical loops. Biol. Cybern. 66, 241–251 10.1007/BF00198477 1540675

[B33] Murray S. O. Kersten D. Olshausen B. A. Schrater P. Woods D. L. (2002). Shape perception reduces activity in human primary visual cortex. Proc. Natl. Acad. Sci. U.S.A. 99, 15164–15169 10.1073/pnas.192579399 12417754 PMC137561

[B34] Norman K. A. Polyn S. M. Detre G. J. Haxby J. V. (2006). Beyond mind-reading: multi-voxel pattern analysis of fMRI data. Trends Cogn. Sci. 10, 424–430 10.1016/j.tics.2006.07.005 16899397

[B35] Peltier S. Stilla R. Mariola E. LaConte S. Hu X. Sathian K. (2007). Activity and effective connectivity of parietal and occipital cortical regions during haptic shape perception. Neuropsychologia 45, 476–483 10.1016/j.neuropsychologia.2006.03.003 16616940

[B36] Pressnitzer D. Hupé J. M. (2006). Temporal dynamics of auditory and visual bistability reveal common principles of perceptual organization. Curr. Biol. 16, 1351–1357 10.1016/j.cub.2006.05.054 16824924

[B37] Rao R. P. N. Ballard D. H. (1999). Predictive coding in the visual cortex: a functional interpretation of some extra-classical receptive-field effects. Nat. Neurosci. 2, 79–87 10.1038/4580 10195184

[B38] Rees G. Kreiman G. Koch C. (2002). Neural correlates of consciousness in humans. Nat. Rev. Neurosci. 3, 261–270 10.1038/nrn783 11967556

[B39] Rieger J. W. Grüschow M. Heinze H. J. Fendrich R. (2007). The appearance of figures seen through a narrow aperture under free viewing conditions: effects of spontaneous eye motions. J. Vis. 7:10 10.1167/7.6.10 17685793

[B40] Rieger J. W. Reichert C. Gegenfurtner K. R. Noesselt T. Braun C. Heinze H. J. (2008). Predicting the recognition of natural scenes from single trial MEG recordings of brain activity. Neuroimage 42, 1056–1068 10.1016/j.neuroimage.2008.06.014 18620063

[B41] Tong F. Meng M. Blake R. (2006). Neural bases of binocular rivalry. Trends Cogn. Sci. 10, 502–511 10.1016/j.tics.2006.09.003 16997612

[B42] Tong F. Nakayama K. Vaughan J. T. Kanwisher N. (1998). Binocular rivalry and visual awareness in human extrastriate cortex. Neuron 21, 753–759 10.1016/S0896-6273(00)80592-9 9808462

[B43] Tong F. Pratte M. S. (2012). Decoding patterns of human brain activity. Annu. Rev. Psychol. 63, 483–509 10.1146/annurev-psych-120710-100412 21943172 PMC7869795

[B44] Ungerleider L. G. Mishkin M. (1982). Two cortical visual systems, in Analysis of Visual Behavior, eds Ingle D. J. Goodale M. A. Mansfield R. J. W. (Cambridge: MIT Press), 549–586

[B45] van Ee R. van Dam L. C. J. Brouwer G. J. (2005). Voluntary control and the dynamics of perceptual bi-stability. Vision Res. 45, 41–55 10.1016/j.visres.2004.07.030 15571737

[B46] Vapnik V. N. (1998). Statistical Learning Theory. New York, NY: John Wiley & Sons

[B47] von Helmholtz H. (1867). Handbuch der Physiologischen Optik. Hamburg: Voss

[B48] Weiskopf N. (2012). Real-time fMRI and its application to neurofeedback. Neuroimage 62, 682–692 10.1016/j.neuroimage.2011.10.009 22019880

[B49] Weiskopf N. Sitaram R. Josephs O. Veit R. Scharnowski F. Goebel R. (2007). Real-time functional magnetic resonance imaging: methods and applications. Magn. Reson. Imaging 25, 989–1003 10.1016/j.mri.2007.02.007 17451904

[B50] Yin C. Shimojo S. Moore C. Engel S. A. (2002). Dynamic shape integration in extrastriate cortex. Curr. Biol. 12, 1379–1385 10.1016/S0960-9822(02)01071-0 12194818

[B51] Zaretskaya N. Anstis S. Bartels A. (2013). Parietal cortex mediates conscious perception of illusory Gestalt. J. Neurosci. 33, 523–531 10.1523/JNEUROSCI.2905-12.2013 23303932 PMC6704896

